# Sex as a modifier of genetic risk for type 1 diabetes

**DOI:** 10.1111/dom.70124

**Published:** 2025-09-18

**Authors:** Hui‐Qi Qu, Hakon Hakonarson

**Affiliations:** ^1^ The Center for Applied Genomics Children's Hospital of Philadelphia Philadelphia Pennsylvania USA; ^2^ Department of Pediatrics, The Perelman School of Medicine University of Pennsylvania Philadelphia Pennsylvania USA; ^3^ Division of Human Genetics Children's Hospital of Philadelphia Philadelphia Pennsylvania USA; ^4^ Division of Pulmonary Medicine Children's Hospital of Philadelphia Philadelphia Pennsylvania USA; ^5^ Faculty of Medicine University of Iceland Reykjavik Iceland

**Keywords:** gene expression, genetic risk, immune cells, sex, type 1 diabetes

## Abstract

Sex differences influence the pathogenesis of type 1 diabetes (T1D), yet most genetic studies have treated sex as a control covariate rather than a dynamic effect modifier. Sex influences immune cell behaviour, including CD4^+^ and CD8^+^ T cell activation, regulatory T cell stability, B cell autoantibody production, dendritic cell priming and monocyte/macrophage inflammation. Underlying mechanisms include hormone‐responsive enhancers, X‐escape gene dosage and sex‐biassed chromatin states, intersecting with T1D‐associated variants to produce sex‐specific immune phenotypes. These insights help explain regional variation in sex ratios of T1D incidence, such as male predominance in high‐risk populations and female excess in low‐risk populations. Biological sex shapes T1D risk across multiple layers, including polygenic load; environmental exposures such as vitamin D deficiency and enteroviral infection; and sex‐specific hormonal, chromosomal and epigenetic influences. An integrative G × E × S (genetic × environmental × sex‐specific) liability‐threshold framework is thus supported. Clinical and translational implications include developing sex‐specific polygenic risk scores, biomarker panels and interventional strategies targeting pathways such as hormone signalling, vitamin D metabolism and the microbiome. Future multi‐omic, longitudinal studies are warranted to test genotype–sex interactions, integrate sex as a core effect modifier and enable precision prevention and treatment of T1D in both males and females.

## 1 INTRODUCTION

Type 1 diabetes (T1D) is an autoimmune disease characterised by the destruction of insulin‐producing pancreatic β‐cells.^1^ This process is orchestrated by a complex network of immune cells.^2^, ^3^ Once initiated, the immune‐mediated β‐cell destruction progresses at an accelerated pace, resulting in lifelong insulin dependence. T1D is widely recognised as a complex genetic disorder, with strong associations observed at the human leukocyte antigen (*HLA*) class II region, which alone accounts for up to 50% of genetic risk.^4^ Specific *HLA‐DQ* haplotypes, particularly the heterozygous combination of DQA1*05:01‐DQB1*02:01 and DQA1*03:0X‐DQB1*03:02, confer the highest known genetic risk for T1D.^5^ This combination encodes trans‐complementing DQ heterodimers with enhanced ability to present β‐cell antigens to autoreactive CD4^+^ T cells, for example, via impaired peptide editing by HLA‐DM.^6^ The *HLA* class I region also modulates T1D susceptibility (e.g., by shaping CD8^+^ T cell responses).^7^ In addition to the *HLA* region, numerous non‐HLA loci have been implicated in T1D through genome‐wide association studies (GWAS).^8^ These include the insulin gene (*INS*), which encodes a primary β‐cell autoantigen, as well as genes involved in immune regulation, such as *PTPN22*, *IL2RA*, *CTLA4*, *IFIH1* and *TYK2*, many of which influence T cell signalling, antigen presentation, regulatory T cell (Treg) stability and cytokine responsiveness.^8^ Collectively, these loci establish a polygenic risk architecture that centres on immune cell function directed against β‐cells. The T1D Genetic Risk Score 2 (T1D‐GRS2), developed by Sharp et al.^5^ based on 67 causal variants, has proven to be a powerful tool for identifying individuals at high risk before the onset of irreversible autoimmunity.

**FIGURE 1:**
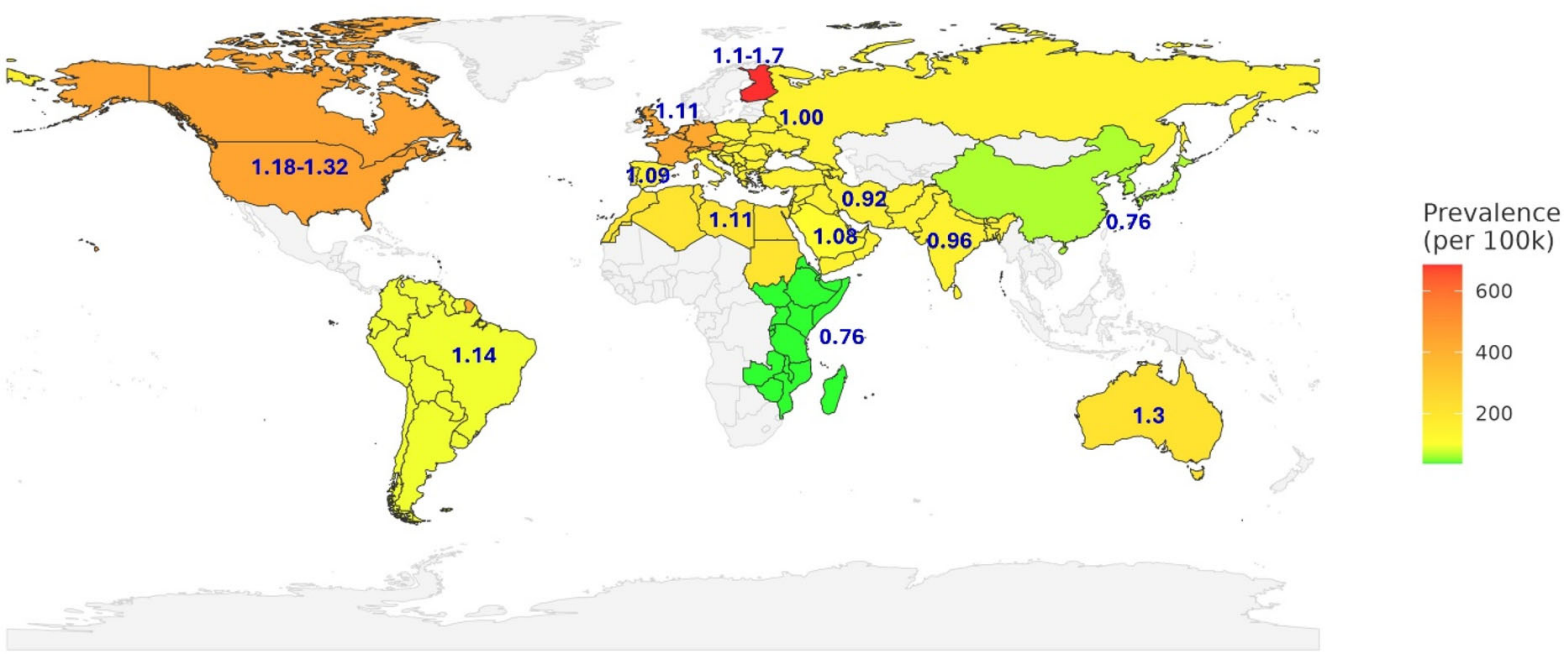
Type 1 diabetes (T1D) prevalence and male‐to‐female incidence ratio by region. Regions are shaded by T1D prevalence (green = low, yellow = mid, red = high) and annotated with the male‐to‐female incidence ratio. Selected countries and regions are shown to demonstrate how sex ratio varies with prevalence. The data included here do not comprehensively represent all world regions. Regions such as the United States and Australia represent ethnically diverse populations. T1D prevalence values are from studies.^9^, ^10^, ^11^, ^12^, ^13^, ^14^, ^15^ Basemap created using the rnaturalearthdata R package.^16^

Accumulating evidence suggests that biological sex modifies the manifestation and impact of genetic risk in T1D, as supported by epidemiological observations (Figure 1). Notably, in high‐incidence European‐ancestry populations, a male predominance emerges during adolescence and persists into adulthood. The Swedish Childhood Diabetes Register (1983–2002) reported annual incidence rates of 16.4 per 100 000 in males versus 8.9 per 100 000 in females (M:F ≈ 1.8).^17^ Similarly, Finnish registry data show that incidence in early childhood is comparable between sexes (boy∶girl ≈1.1 overall), rising to approximately 1.7 by early adolescence.^9^ As reviewed by Maahs et al., regions with high T1D incidence (predominantly European‐origin) tend towards male excess, whereas lower‐incidence regions (non‐European) more often exhibit a female predominance.^18^ In the United States, male predominance becomes apparent by about age 10 and persists into adulthood, with reported M:F incidence ratios ranging from approximately 1.18–1.32 across different studies.^10^, ^19^ These observations raise the possibility that sex differences in T1D incidence arise from complex interactions involving both intrinsic biological factors and external influences, shaped by developmental timing, immune status and environmental context.

Beyond these broad patterns, sex‐specific incidence varies by age, geography and time. In most populations, sex ratios are comparable in early childhood but diverge at puberty, with male predominance emerging during adolescence and persisting into adulthood in high‐incidence regions.^9^, ^17^ By contrast, in lower‐incidence settings such as East Asia and Eastern Africa, female predominance has been observed, suggesting that baseline genetic and environmental contexts modulate the direction of sex bias.^18^ Temporal trends further reveal that in Scandinavia and other high‐incidence cohorts, male excess has become more pronounced over recent decades, coinciding with rising background incidence, whereas female predominance persists in lower‐incidence populations.^11^ Together, these observations underscore that age, ancestry and secular change shape sex ratios in T1D and should be explicitly considered in mechanistic models.

Sex‐specific immune signatures in T1D, such as the distinct autoantibody repertoires and immunophenotypes observed in females, suggest underlying differences in immune tolerance and environmental exposures.^20^ However, conventional genetic risk models often treat sex merely as a covariate and fail to model it as a biological modifier. This analytic choice may obscure important sex‐specific disease mechanisms and limit the interpretability of genetic associations. Understanding how sex modifies genetic risk, particularly within immune cells that are central effectors of β‐cell autoimmunity, is valuable for refining our knowledge of T1D pathogenesis. In this review, we critically evaluate how biological sex interacts with genetic variation at the cellular and molecular levels, as well as through population‐level factors, to influence T1D susceptibility, offering a foundation for improved risk prediction.

## 2 SEX DIFFERENCES IN IMMUNE CELL FUNCTION AND T1D

Sex modifies multiple immune compartments that drive β‐cell autoimmunity in T1D. CD4^+^ T cells restricted by high‐risk *HLA* class II alleles initiate and sustain insulitis by providing help to CD8^+^ T cells and B cells, while CD8^+^ T cells restricted by class I alleles such as *HLA*‐B*39:06 and HLA‐A*24:02 directly mediate β‐cell killing.^7^, ^21^ Tregs contribute to tolerance, with oestrogen signalling and *FOXP3* dosage influencing their frequency and stability.^22^ B cells shape risk through autoantibody responses that differ by sex at disease onset.^23^ Antigen‐presenting cells (APCs), including dendritic cells and macrophages, amplify or restrain autoreactivity under the influence of hormones and sex chromosome complement.^24^ These cell type‐specific effects are summarised in Table 1, with extended discussion in Supplementary Text 1.

**TABLE 1:** Sex effects on immune cell function in type 1 diabetes (T1D).

Cell type	Sex hormones	Sex chromosomes	Epigenetics	Overall sex effect
CD4^+^ helper T cells	Oestrogen ↑ interleukin‐12 (IL‐12)‐STAT4/T‐bet → Th1/Th17 bias^22^; androgen suppresses Th1 axis^25^	X‐escape genes may amplify female T cell receptor (TCR) signalling^26^	Epigenetic mechanisms shaping helper skewing^27^	Females: stronger Th1/Th17 responses → higher autoimmunity in non‐obese diabetic (NOD) mice^28^; males: androgen‐mediated protection, but higher basal Th1∶Th2 ratio in humans^22^
CD8^+^ cytotoxic T cells	Androgen receptor (AR) signalling ↓ *IFNG*/*GZMB*, ↑ programmed cell death protein 1 (PD‐1) exhaustion^29^, ^30^; oestrogen effects on activation/trafficking during puberty^22^	X/Y complement influences baseline responsiveness^31^	Epigenetic factors shape effector versus exhaustion fate^31^	Males: AR restraint dampens β‐cell cytotoxic T lymphocytes (CTLs)^29^, ^30^; females: higher cytotoxic gene expression but faster exhaustion^32^
Regulatory T cells (Tregs)	Oestrogen ↑ FOXP3 induction & stability^31^, ^33^; androgens variably affect Treg frequency^34^, ^35^	*FOXP3* on X chromosome; XCI/escape patterns may tweak expression^22^	Epigenetic regulators maintain *FOXP3* stability^24^; hypomethylation + oestrogen response elements (EREs) enhance oestrogen sensitivity^36^, ^37^	Females: oestrogen‐driven higher Treg induction^31^, ^33^; post‐pubertal males may exhibit stronger Treg function^34^, ^35^
B cells	Oestrogen ↑ maturation, class‐switch recombination & somatic hypermutation^38^	X‐linked signalling genes modulate B cell receptor (BCR) thresholds^31^; mosaicism biases autoreactivity^39^	Epigenetic marks govern germinal centre dynamics^31^	Females: stronger autoantibody production and faster epitope spreading^40^, ^41^; males: puberty‐linked insulin autoantibody peak^23^
Dendritic cells (DCs)	Oestrogen ↑ DC maturation, major histocompatibility complex (MHC) II and IL‐12 production^42^; androgen ↓ DC maturation & pro‐inflammatory cytokines^43^; Testosterone paradoxically ↑ IL‐12 in vitro^23^	X/Y regulators likely influence subset distribution^31^	Epigenetic control of DC identity and cytokine profiles^31^	Females: stronger priming of autoreactive T cells^42^; males: reduced DC‐driven activation but context‐dependent^43^
Monocytes/macrophages	Oestrogen ↑ Toll‐like receptor (TLR)‐driven tumor necrosis factor‐alpha (TNF‐α)/IL‐6^44^, ^45^; androgen attenuates TLR responses^46^	Sex chromosome complement affects differentiation and polarisation^47^; loss of redundancy in males increases variant impact^39^, ^48^	Epigenetic programming shapes cytokine output and phagocytosis^49^; chromatin state determines hormone–SNP interaction^50^	Females: heightened innate inflammation → accelerated insulitis^44^, ^45^; males: M2‐skewing with elevated Bcl‐2 and transforming growth factor‐beta 1 (TGF‐β1) promotes survival but impairs apoptotic β‐cell clearance^51^

Findings from T1D‐prone NOD mice and human cohorts indicate that sex alters both effector and regulatory balance across the immune system. In NOD mice, oestrogen‐driven amplification of IL‐12‐STAT4‐T‐bet signalling enhances Th1 and Th17 polarisation in females, whereas androgens suppress this axis and confer protection.^22^ In humans, strong *HLA* class II risk often establishes autoreactive CD4^+^ responses before puberty, when sex steroid levels have not yet diverged. Men display a higher Th1:Th2 cytokine ratio than women, which can amplify β‐cell‐directed responses,^22^ while early‐life exposures such as viral infections or microbiome perturbations interact with these baseline differences to shape trajectories towards disease.^52^ Together, these observations suggest that sex modifies the tempo and severity of islet autoimmunity rather than acting as a primary determinant of risk.

## 3 MECHANISTIC MODELS OF SEX–GENOTYPE INTERACTION IN IMMUNE CELLS

Several interacting layers explain how sex modifies genetic risk in immune cells relevant to T1D. Sex hormones engage nuclear receptors that bind enhancer and promoter motifs near disease‐associated variants, producing allele‐ and context‐specific transcriptional effects. Oestrogen‐responsive elements at *IL2RA* or *FOXP3* can strengthen tolerance pathways, whereas androgen receptor signalling restrains CD8^+^ effector programmes and alters myeloid cytokine production.^24^, ^29^

Independent of hormones, sex chromosome complement introduces dosage differences. Escape from X‐chromosome inactivation (XCI) leads to biallelic expression of immune genes such as *TLR7*, while skewed inactivation can bias immune cell compartments towards one allele.^39^ These dosage effects interact with autosomal variants, magnifying or mitigating their functional impact.

Sex‐biassed epigenetic architecture also plays a role. Chromatin accessibility and DNA methylation differ between male and female immune cells,^53^ and several of these regulatory differences overlap loci implicated in T1D, including *IL2RA*, *FOXP3* and *INS*.^54^ Because hormone receptors preferentially bind pre‐opened chromatin,^36^ epigenetic state dictates when and in whom hormone–genotype interactions can occur, thereby linking sex differences in chromatin landscape to functional variation at T1D risk genes.

Timing is critical. T1D often develops in childhood, when hormone levels are low and unstable, meaning that sex effects may be insufficient to prevent initiation of autoimmunity. Later increases in sex steroids may modulate disease progression but cannot erase autoreactivity once established. Variability in androgen receptor signalling and Y‐linked immunoregulatory factors further shapes outcome, contributing to the heterogeneous sex bias observed across populations.^55^ Expanded mechanistic details are provided in Supplementary Text 2.

## 4 LOCUS‐SPECIFIC MECHANISTIC MODELS IN T1D

To directly connect sex‐modified immune regulation to T1D pathogenesis, we highlight loci with established causal roles in disease risk, including *HLA* class II, *INS* VNTR, *IL2RA*, *CTLA4* and *CLEC16A*. These genes are repeatedly associated with T1D across populations, and each demonstrates interaction with sex‐specific mechanisms such as hormone‐responsive enhancers,^56^ X‐linked dosage,^57^ or epigenetic regulation.^54^

### 4.1 *HLA* Class II

In T1D, the HLA‐DQA1*05:01‐DQB1*02:01 and DQA1*03:0X‐DQB1*03:02 heterodimers confer the most substantial genetic risk.^5^ The former is also known as *DQ2.5*, a subtype of *DQ2*, and DQA1*03:01‐DQB1*03:02 as *DQ8*.^4^ The abundance of these class II molecules on APCs is orchestrated by class II major histocompatibility complex transactivator (*CIITA*), whose promoter IV (pIV) integrates signals from both endocrine and cytokine pathways.^58^ In cells expressing oestrogen receptor α (ERα), 17β‐estradiol (E₂) modulates interferon‐gamma (IFN‐γ)‐induced *CIITA* pIV activity in a context‐dependent manner. Notably, in ERα^+^ breast cancer cells engineered to model antigen‐presenting features, E₂ treatment attenuates IFN‐γ‐driven *CIITA* transcription and subsequent *HLA‐DR* expression, which suppression can be fully reversed by the endoplasmic reticulum (ER) antagonist or ESR1 knockdown, demonstrating direct ERα engagement at *CIITA* pIV^58^ (relevance to pancreatic APCs remains to be validated). Further, the steroid receptor coactivator 1 (SRC‐1) is recruited to the *CIITA* complex upon IFN‐γ stimulation and can relieve oestrogen‐mediated inhibition, underscoring nuanced crosstalk between hormone receptors and IFN‐γ signalling at this locus.^59^

Though *CIITA* itself resides on chromosome 16, X‐linked innate sensors impart a chromosome‐level influence on class II regulation. *TLR7* escapes XCI in female B cells, monocytes and plasmacytoid dendritic cells, resulting in biallelic expression and amplified *TLR7*‐driven type I interferon production. Biallelic *TLR7* ^+^ pDCs from women secrete significantly more interferon‐alpha/beta (IFN‐α/β) than monoallelic cells, thereby priming downstream IFN‐γ pathways and potentially enhancing *CIITA*‐mediated *HLA* class II expression in a female‐biassed fashion (direct *TLR7* → *CIITA* signalling remains unproven).^60^, ^61^

Chromatin accessibility profiling of human haematopoietic precursors reveals extensive sex‐specific differences in enhancer architecture that precede pubertal hormonal maturation. In combined single‐cell assay for transposase accessible chromatin sequencing (ATAC‐seq) and cellular indexing of transcriptomes and epitopes sequencing (CITE‐seq) analyses of 4‐ to 5‐month‐old human postnatal thymocytes, female CD3+ double positive cells (DPs) exhibit constitutively greater accessibility at *CIITA* pIV than their male counterparts, which permits the immediate recruitment of ERα and STAT1 complexes upon IFN‐γ stimulation.^62^ This epigenetic priming endows female APCs with a more efficient *CIITA* transcriptional response to subsequent cytokine or steroidal cues, ultimately facilitating enhanced *HLA* class II expression.

Collectively, these layers converge to amplify *HLA* class II output in females: escape from X inactivation heightens baseline cytokine signalling (chromosomal), pre‐opened chromatin at *CIITA* pIV permits rapid transcription factor recruitment (epigenetic) and ERα modulation fine‐tunes promoter activity in concert with IFN‐γ‐activated complexes (hormonal). This integrated framework explains why *DQ2*/*DQ8* surface levels and antigen presentation capacity can be intrinsically elevated in women under given genetic and inflammatory conditions. The reduced T1D risk in females may reflect enhanced tolerogenic mechanisms, notably oestrogen‐driven expansion and functional augmentation of FoxP3^+^ Tregs.^63^

### 4.2 *INS* VNTR

Genetic variation at the *INS* VNTR locus exerts a profound influence on central tolerance by modulating insulin expression in thymic epithelial cells (TEC). In seminal studies, Pugliese et al. and Vafiadis et al. showed that the short class I VNTR haplotype is associated with a two‐ to three‐fold reduction in insulin mRNA levels in human thymus compared to protective class III alleles, implicating quantitative differences in thymic insulin transcripts as a key determinant of autoreactive T cell deletion.^64^ Increased thymic insulin expression promotes more efficient negative selection of insulin‐reactive T cells, thereby bolstering central tolerance.

ERα is abundantly expressed in TEC and governs thymic architecture and gene programmes: ERα‐deficient mice fail to undergo oestrogen‐driven thymic involution and display altered TEC composition, supporting a role for fluctuating oestrogen levels in shaping *INS* VNTR‐dependent transcriptional output.^65^

Sex‐specific differences in thymic architecture can influence the organisation and function of central tolerance compartments. Dumont‐Lagacé et al. reported that adult male mice accumulate more cortical TECs relative to females, implying that female thymuses maintain proportionally expanded medullary compartments, where insulin is presented to developing thymocytes, modulating the efficiency of negative selection.^66^ Complementary work demonstrates that sex steroids exert pervasive effects on TEC biology, with oestrogens and androgens differentially regulating TEC subset distributions and thymic involution kinetics, indirectly tuning the medullary environment for insulin tolerance induction.^65^

Epigenetic regulation at the *INS* VNTR locus is indirectly shaped by sex‐specific expression of the autoimmune regulator *AIRE*, which recruits chromatin‐remodelling machinery to activate tissue‐restricted antigens. Dragin et al. demonstrated that oestrogen exposure increases methylation of the *AIRE* promoter in female TEC, resulting in reduced *AIRE* mRNA and protein levels compared to males.^57^ Sabater et al. found that although class III VNTR alleles are associated with elevated thymic *INS* transcripts, inter‐individual variability in *INS* expression aligns more closely with *AIRE* abundance than VNTR genotype alone,^67^ indicating that sex‐driven fluctuations in *AIRE* supersede allelic effects at the INS promoter. Sparks et al. further showed that *AIRE*'s plant homeodomain (PHD) type zinc fingers (histone mark‐reading modules) and Leucine–X–X–Leucine–Leucine motif (LXXLL) motifs are essential for *AIRE*‐dependent activation of the *INS* VNTR in human TEC, highlighting the centrality of *AIRE*‐mediated chromatin remodelling at this locus.^64^

Together, these studies suggest that female‐biassed, oestrogen‐mediated epigenetic modulation of *AIRE* fine‐tunes thymic insulin availability, favouring efficient central tolerance in females, which may contribute to the lower incidence of T1D in women.

### 4.3 *IL2RA* (CD25)

Interleukin‐2 receptor α (CD25), encoded by *IL2RA* on chromosome 10p15, is critical for T cell proliferation and Treg homeostasis. Large‐scale fine mapping has localised T1D association signals to two distinct IL2RA regions spanning intron 1 and the 5′ promoter, and risk genotypes correlate with lower circulating soluble IL‐2RA levels, suggesting that impaired IL‐2 responsiveness predisposes to autoimmunity, for example, the two T1D‐GRS2 markers rs61839660 and rs41295121 in the *IL2RA* locus.^68^, ^69^ A functional intronic single nucleotide polymorphism (SNP), rs12722489 [in low linkage disequilibrium (LD) with the two *IL2RA* T1D‐GRS2 markers with *r* ^2^ < 0.02], lies within an oestrogen‐responsive element: the protective A allele enhances ERα binding and increases enhancer activity in human Treg‐like cells, whereas the risk G allele abolishes this effect, linking sex hormones directly to IL2RA transcriptional control.^56^ Moreover, methylation profiling in paediatric lymphocyte subsets shows that rs12722495 (in high LD with rs61839660 with *r* ^2^ = 0.936) associates with promoter CpG hypomethylation in CD4^+^ T cells from girls, but not boys, concurrently with elevated IL2RA mRNA, indicating a sex‐specific epigenetic quantitative trait effect.^53^ Thus, oestrogen‐driven enhancer activity works in concert with genotype‐dependent methylation patterns within the chromosomal regulatory context to produce sex‐specific IL2RA expression. Together, these effects may augment IL2RA levels, strengthen IL‐2 signalling and stabilise Treg‐mediated tolerance in females.

### 4.4 CTLA4

The CTLA‐4 checkpoint molecule, encoded on chromosome 2q33, is a pivotal brake on T cell activation. A common 3′ untranslated region (UTR) polymorphism, CT60 (rs3087243), correlates with altered splicing and mRNA stability: carriers of the risk G allele exhibit reduced soluble CTLA‐4 isoform transcripts and heightened susceptibility to T1D and other autoimmune disorders. Systemic oestrogen exposure, as shown by Polanczyk et al., expands the FoxP3^+^ Treg compartment (CTLA‐4^+^ cells), thereby reinforcing peripheral tolerance in females.^63^

Overall, these gene‐specific mechanisms tend to operate before and during the initial β‐cell antigen encounter well before puberty and clinical onset, and uniquely enhance female protection in T1D. In contrast, autoimmune diseases that arise later or target non‐β‐cell antigens do not benefit from this coordinated enhancement of insulin‐specific central and regulatory tolerance.

### 4.5 CLEC16A

*CLEC16A*, an E3‐ubiquitin ligase on chromosome 16p13.13, is essential for mitophagy in both β‐cells and immune cells, and *CLEC16A* risk variants compromise mitochondrial turnover and antigen‐processing pathways, contributing to T1D pathogenesis.^70^, ^71^ Sex hormones likewise shape immune autophagy: oestrogen promotes autophagy in plasmacytoid dendritic cells and macrophages, whereas androgen's effects remain understudied. Androgen receptor (AR) inhibition alleviates inflammation in experimental autoimmune myocarditis by enhancing autophagy, especially in macrophages.^72^ This male AR‐driven autophagy suppression may diminish *CLEC16A*‐dependent mitophagy in β‐cells and APCs.

Together, loci such as *IL2RA*, *FOXP3*, *INS* VNTR, *CTLA4* and *CLEC16A* represent immediate candidates for longitudinal studies designed to test sex‐specific effects. These regions illustrate how endocrine modulation, X‐linked dosage and epigenetic state intersect with genetic variation, and they provide a concrete starting point for identifying biomarkers and refining predictive models.

## 5 Β‐CELL‐INTRINSIC SEX DIFFERENCES

Emerging evidence indicates that pancreatic β‐cells themselves exhibit sex‐specific functional and stress‐response phenotypes that shape susceptibility to autoimmune attack, with female β‐cells displaying remarkably enhanced protective mechanisms. In mouse and human β‐cells, ERα signalling mediates cytoprotective effects by preserving mitochondrial integrity and suppressing endoplasmic reticulum stress via downregulation of apoptotic effectors such as C/EBP homologous protein (CHOP) and upregulation of chaperones including binding immunoglobulin protein (BiP).^73^, ^74^ Transcriptomic and proteomic analyses of islets from female donors reveal higher baseline expression of unfolded protein response and antigen‐presentation genes, including *HLA* class I molecules *HLA‐A* and *HLA‐B*, and greater resilience to chemically induced endoplasmic reticulum stress, with female islets maintaining glucose‐stimulated insulin secretion under thapsigargin treatment while male islets show marked secretory failure.^75^, ^76^ In parallel, rapid non‐genomic oestrogen signalling through the G protein‐coupled oestrogen receptor (GPER) activates PI3K/Akt and extracellular signal–regulated kinase (ERK) pathways, mobilises intracellular calcium and enhances insulin release at both low and high glucose in MIN6 cells and isolated islets, effects that are abolished by GPER antagonism or genetic knockout.^77^ Oestrogen also activates Nrf2‐dependent antioxidant pathways in β‐cells, reducing oxidative stress‐induced apoptosis.^78^

Together, these β‐cell‐intrinsic sex differences in stress resilience, antigen‐presentation capacity and hormone‐driven signalling create a distinct threshold for survival under autoimmune attack and interact with systemic sex‐hormone profiles to influence the timing and penetrance of clinical T1D onset. Conversely, AR signalling in β‐cells may influence oxidative stress resilience in males, representing an important area for further study.^79^

## 6 MECHANISMS UNDERLYING REGIONAL SEX DIFFERENCES IN T1D RISK

**FIGURE 2:**
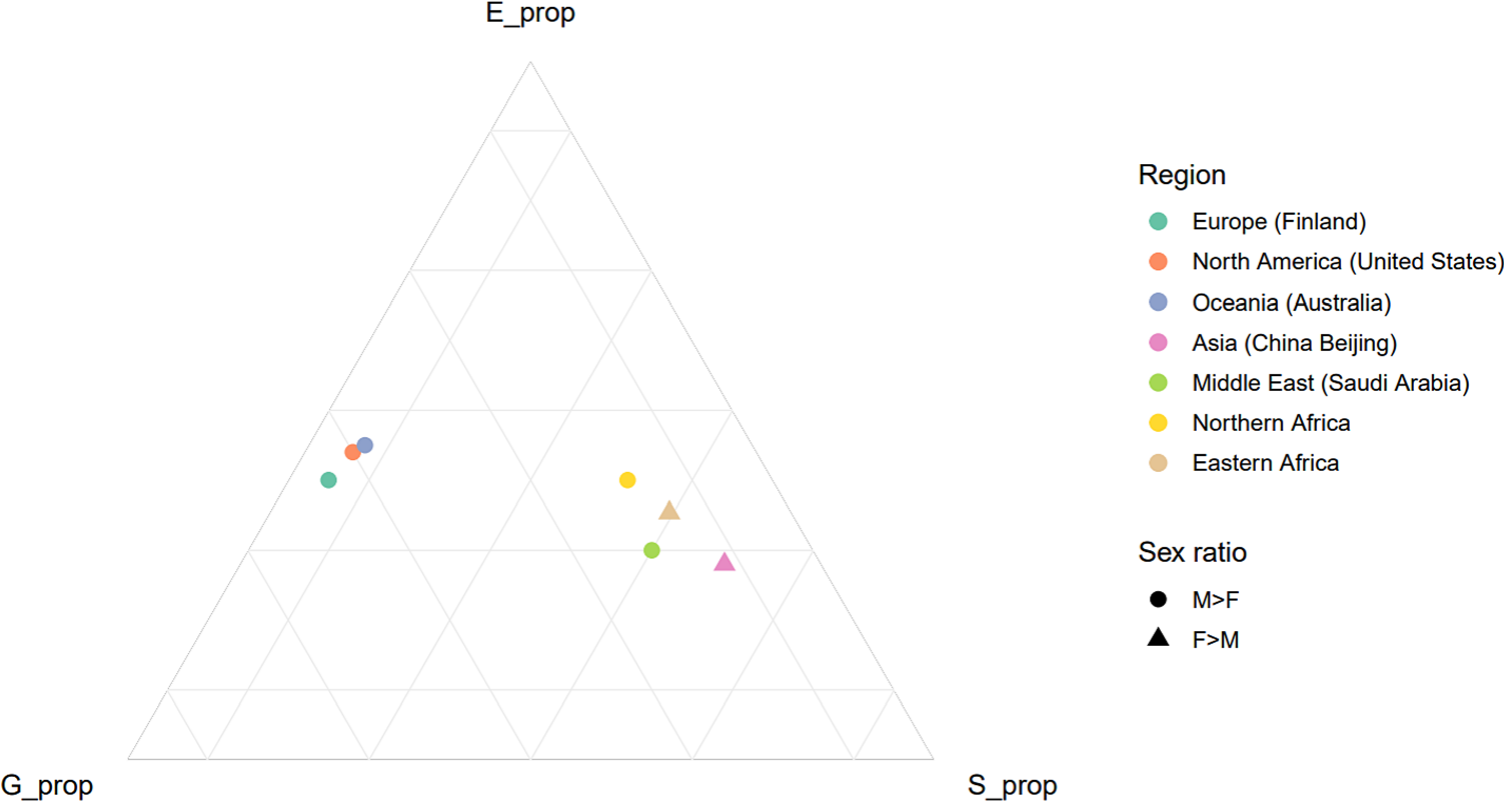
Conceptual ternary model of genetic (G), environmental (E) and sex‐specific (S) contributions across regions. Each marker denotes a region and its relative G:E:S proportions summing to 100%. If G is the engine's horsepower and E the accelerator pedal, then S is the transmission gearbox, whose gear selection dictates how efficiently that horsepower is converted into acceleration, amplifying or damping the same pedal input based on the gear. G:E:S values are illustrative only and not based on measured data.

While earlier sections examined cellular and molecular mechanisms, it is also important to explore how these processes manifest within broader population contexts. Epidemiological data show geographic variation in sex ratios of T1D incidence. Male predominance (M > F) is typical in the high‐incidence regions that span Northern Europe and North America, whereas female excess (F ≥ M) is reported in many low‐incidence areas, including East Asia and Eastern Africa (Figure 1).^18^ These regional contrasts reflect the intersection of sex‐specific biology with ancestry‐linked genetics and diverse environmental and modulatory factors (Figure 2, conceptual model).

Future studies should explicitly dissect G × E × S interactions by validating associations, quantifying effect sizes and testing predictive value in longitudinal cohorts. On the genetic side (G), this includes assessing whether high‐risk *HLA* haplotypes and polygenic risk scores (PRS) show sex‐biassed penetrance and estimating how much of the variance in liability they explain in males versus females across diverse ancestries. On the environmental side (E), exposures such as vitamin D deficiency, enteroviral infection and microbiome perturbations should be modelled for sex‐dependent effects, with formal interaction terms to determine whether they modify genetic risk differently in boys and girls. On the sex‐specific side (S), pubertal timing, circulating hormone trajectories and sex chromosome complement must be integrated into these analyses to quantify their modifying influence on genetic susceptibility.

Age strata, ethnicity and temporal shifts further nuance regional sex differences in T1D. US SEARCH data indicate that sex ratios are not uniform across ethnic groups, with patterns differing between non‐Hispanic White and African American youths.^10^ In Scandinavia, long‐term registry analyses show that male excess has intensified as overall incidence has risen.^17^ By contrast, in East Asia, where incidence is increasing from a low baseline, a female predominance persists.^15^ These observations suggest that the interaction of sex with genetic architecture, environment and developmental timing evolves dynamically as populations undergo epidemiological transition.

### 6.1 Population genetic architecture

Northern Europeans carry the highest frequencies of the *HLA‐DQ* haplotypes that dominate T1D risk.^4^ In such high‐load contexts, the effect of high‐risk alleles may overwhelm the generally modest immunosuppressive action of androgens, allowing a male excess to emerge. East‐Asian populations, in contrast, have lower frequencies of these haplotypes and a higher prevalence of protective alleles (e.g., HLA‐DRB1*15 variants),^4^ so baseline genetic load is smaller and female‐biassed autoimmunity may emerge more readily. Y‐chromosome haplogroups prevalent in Northern Europe (e.g., I1, R1b) exhibit distinct male‐specific region of the Y chromosome (MSY) gene content and regulatory‐element landscapes compared to East‐Asian haplogroup O lineages, with haplogroup I1 associated with altered expression of Y‐linked genes such as *UTY* and *PRKY* in immune cells,^80^ suggesting potential modulation of androgen‐receptor‐dependent transcription in immune cells.^81^ Frequencies of X‐linked escape genes vary across populations, affecting the extent of biallelic expression in females^82^ and might augment innate immune signalling.

### 6.2 Latitude, ultraviolet B (UV‐B) and vitamin D signalling

**FIGURE 3:**
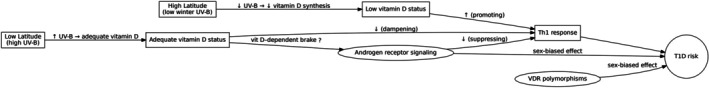
Vitamin D‐dependent modulation of Th1 response and sex‐biassed type 1 diabetes (T1D) risk across a latitudinal gradient. Latitudinal differences (in UV‐B exposure, as well as skin pigmentation, clothing, outdoor behaviour, dietary intake and supplementation and other factors [e.g., air pollution]) drive vitamin D status and thereby modulate Th1‐mediated immune responses (among other pathways), with low vitamin D promoting Th1‐driven autoimmunity and adequate vitamin D damping it. Androgen receptor signalling, a potential vitamin D‐influenced brake, which may interact with vitamin D signalling, and *VDR* polymorphisms act as modifiers, potentially with sex‐specific effects, that converge on the smaller circle endpoint of T1D risk.

T1D incidence follows a classic north–south gradient that parallels winter UV‐B exposure.^83^ Vitamin D status is sexually dimorphic: men tend to show a stronger inverse association between serum 25‐OH‐D and insulin resistance/autoimmunity than women, and *VDR* polymorphisms display sex‐biassed effects on T1D risk.^84^ In high‐latitude settings, men tend to have lower serum 25‐OH‐D levels than women, and this greater vitamin D deficiency can exacerbate their higher baseline Th1 bias. Near the equator, more abundant UV‐B narrows these hormonal gaps, and any female‐predominant humoral immunity can dominate (Figure 3).

### 6.3 Enteroviral and other infectious exposures

Enterovirus infections are the most consistently linked environmental trigger of islet autoimmunity. Maternal enterovirus infection during pregnancy is associated with an increased T1D risk in male offspring.^85^ Postnatal enterovirus infections in early childhood likewise elevate T1D risk.^86^ Regions with intense enterovirus circulation (e.g., Scandinavia) might accrue a higher male burden, whereas countries where early‐life viral exposure is less sex skewed show flatter or reversed ratios.

### 6.4 Microbiome, diet and obesity transitions

Sex‐dependent gut‐microbiota configurations influence systemic autoimmunity in NOD mice; transferring male microbiota to females lowers their diabetes incidence by ~60%.^87^ Human studies are still sparse, but diet‐driven microbiome shifts and rapid nutritional transitions (e.g., westernisation in East Asia) may reshape sex‐specific immune education differently across populations. Rising childhood obesity, which advances pubertal timing, particularly in girls,^88^ may amplify early oestrogen surges and temporarily strengthen B cell and Th17 activity, contributing to the female predominance noted in modernising Asian and Middle‐Eastern societies.

### 6.5 Pubertal timing and sex‐hormone trajectories

The age window in which β‐cell autoimmunity first accelerates overlaps the peri‐pubertal surge of gonadal steroids. Girls worldwide now reach thelarche/menarche 6–12 months earlier per decade in many cohorts, whereas male pubertal onset has shifted less dramatically.^89^ Earlier oestrogen exposure can transiently intensify oestrogen‐responsive enhancers (e.g., at *IL2RA*, *FOXP3*), potentially tipping borderline‐risk girls into clinical diabetes in regions where childhood incidence is still low,^90^ though longitudinal studies directly linking pubertal timing to T1D onset are lacking. In high‐incidence Northern Europe, by contrast, autoantibody seroconversion often precedes puberty, and later‐acting androgen protection becomes the distinguishing variable, again favouring a male excess.

## 7 T1D RISK MODELLING AND CLINICAL TRANSLATION

### 7.1 An integrative threshold model

Building on the sex‐specific mechanisms described above, we propose that clinical T1D onset occurs when an individual's cumulative liability, comprising inherited *HLA* and non‐*HLA* risk alleles (G), external triggers such as vitamin D deficiency and enteroviral exposure (E) and sex‐geared factors like hormone milieu, X/Y dosage and microbiome composition (S), exceeds a critical threshold. In high‐incidence populations (e.g., Northern Europe), heavy *HLA* risk load and intense environmental provocation mean that modest androgen‐mediated immune dampening in boys is insufficient to prevent many males from crossing the threshold first, yielding a male excess. In contrast, low‐load regions (e.g., East Asia), where protective *HLA* alleles predominate and environmental insults are milder, have more boys below the liability threshold; consequently, even modest female‐biassed influences can tip proportionally more girls over that threshold. By integrating G × E × S into a unified liability model, this framework accounts for regional sex ratio differences and assesses T1D risk as a function of genetic variation, dynamic environmental exposures and sex‐related trajectories.

To apply this framework in risk prediction, sex should be treated as an effect modifier rather than simply stratifying by cohorts. Empirical sex × PRS interactions have been documented.^91^ Sex‐specific PRS can be derived by using GWAS summary statistics from male‐only and female‐only analyses, as demonstrated by enriched sex‐differentiated genetic associations at hormone‐responsive loci.^92^ Dynamic, longitudinal biomarkers, such as circulating estradiol or testosterone concentrations and sex‐specific DNA methylation marks at *IL2RA* and other T1D loci, can be included as covariates with interaction terms to capture continuous variation in the S component.^93^ Machine‐learning approaches, including tree‐based methods like gradient boosting machines and random forests, can automatically detect complex, nonlinear sex × genotype interactions, while penalised‐regression models (e.g., elastic net) can manage high‐dimensional interaction feature sets when those interaction terms are pre‐specified.^94^ Time‐to‐event models augmented with time‐varying covariates, such as pubertal status or serial hormone measurements, enable dynamic modelling of genetic risk trajectories across developmental windows in each sex.^95^ These modelling strategies may enable unified, interpretable and biologically grounded risk prediction frameworks, provided that current gaps in longitudinal, sex‐annotated multi‐omic cohorts, standardised pubertal and hormonal phenotyping and transparent modelling workflows prioritising interpretability are addressed.

### 7.2 Clinical and translational implications

Recognising sex as a dynamic modifier, rather than a passive covariate, of T1D risk has immediate implications for prediction, monitoring and prevention (Box 1). One practical step is to enhance existing clinical prediction algorithms, such as the T1D‐GRS2, by incorporating sex‐specific PRS with genome‐wide markers.^96^ This approach moves beyond simple cohort stratification and instead acknowledges sex as an active biological modifier of genetic risk. This is particularly critical in regions and populations with pronounced sex‐modifying effects. A parallel priority is to focus research on sex × PRS interactions in T1D. By calibrating risk scores separately for boys and girls, especially across different age windows around puberty, clinicians can improve the positive predictive value of PRS/Genetic Risk Score for presymptomatic autoantibody screening programmes in high‐risk cohorts. Longitudinal validation of such sex‐specific risk algorithms in large prospective cohorts will be essential for their clinical translation.

BOX 1 **Key future directions for sex‐modified T1D research and translation**

1. *Risk prediction*

- Develop and validate sex‐specific PRS.
- Integrate pubertal timing and circulating hormone measurements into longitudinal prediction models.

2 *Biomarker development*

- Validate sex‐modified genome‐wide markers (including PRS features) in longitudinal cohorts, including established T1D loci (*INS* VNTR, *IL2RA*, *CTLA4* and *CLEC16A*).
- Evaluate sex‐specific epigenetic markers, such as methylation at *IL2RA* and *FOXP3*, as predictive tools.

3 *Clinical trials*

- Design prevention studies incorporating sex‐tailored interventions (e.g., androgen supplementation in boys, selective oestrogen receptor modulators in girls).
- Reanalyze completed prevention trials with sex stratification to detect overlooked efficacy signals.

4 *Therapeutic targets*

- Investigate hormone pathways (androgen receptor, oestrogen receptor, GPER) and vitamin D–sex interactions as modifiable risk factors.
- Explore microbiome–sex interactions as interventional targets.

5 *Cohort infrastructure*

- Encourage sex‐stratified analyses in genetic and immunology studies of T1D.
- Prioritise longitudinal cohort designs that capture pubertal status, circulating sex hormone levels and sex chromosome complement.

Further biomarker discovery and validation may need to account for sex‐biassed immune signatures. Panels of cytokines, autoantibody titers or T cell phenotypes that predict progression in males may differ from those in females. Establishing sex‐stratified reference ranges and testing whether predictive accuracy improves when these immune measures are integrated with dynamic endocrine markers such as estradiol, testosterone or pubertal staging would provide a more precise and individualised assessment of risk. In addition to immune measures, many genome‐wide markers identified through our sex‐stratified GWAS^96^ remain to be systematically evaluated for sex‐specific predictive value in longitudinal cohorts. In that study, we identified >200 sex‐specific SNPs, pinpointed candidate genes with sex‐differential expression, and showed that sex‐specific PRS outperform standard PRS. Priority candidates include genetic and epigenetic markers at established T1D loci such as *INS* VNTR (*AIRE*‐dependent regulation of thymic insulin), *IL2RA* (sex‐dependent enhancer activity), *CTLA4* (sex‐modified checkpoint control) and *CLEC16A* (sex‐influenced autophagy pathways). Epigenetic biomarkers such as *IL2RA* methylation and *FOXP3* enhancer accessibility represent additional candidates for sex‐aware biomarker development.

Therapeutically, several promising directions emerge from sex‐aware mechanistic insights. Future trials might explore whether low‐dose androgen supplementation in peripubertal boys with multiple autoantibodies could enhance physiological androgenic restraint and delay clinical onset. In girls, selective oestrogen receptor modulators may help temper Th1/Th17 polarisation while preserving normal development.^97^ Similarly, sex‐specific optimisation of adjunctive therapies, such as vitamin D repletion, enteroviral vaccination or microbiome modulation, should be evaluated in randomised studies powered to detect differential efficacy by sex.^98^ Retrospective reanalysis of completed prevention studies, including oral insulin, vitamin D supplementation and teplizumab, may further reveal sex‐specific efficacy signals that were previously overlooked and provide immediate guidance for future trial design.

Moving forward, future research would benefit from systematically modelling G × E × S interactions using longitudinal, multi‐omic cohorts (e.g., TEDDY,^99^ TrialNet^100^), integrating sex‐stratified genomic, epigenomic, transcriptomic, proteomic, metabolomic and microbiome data alongside endocrine and immunophenotypic trajectories from birth through adolescence. A specific priority will be to validate whether GWAS‐identified loci with sex‐specific effects show sex‐biassed penetrance, to quantify how environmental exposures such as vitamin D deficiency or enteroviral infection differentially modify these genetic risks in boys and girls, and to determine whether sex chromosome complement further improves the predictive value of polygenic risk models. Large consortia can support rigorous testing of sex‐by‐genotype interactions and epigenetic sex differences at T1D loci and develop in vitro and in vivo models (e.g., humanised immune systems in mice) that recapitulate human sex chromosome complements and hormone environments. These combined efforts will provide the empirical foundation needed to test sex‐specific prediction models and preventive interventions, ensuring that mechanistic insights are systematically advanced towards clinical application.

In conclusion, T1D emerges once cumulative susceptibility crosses a single clinical threshold, while genetic, environmental and sex‐specific factors continually push individuals closer to or further from that point. Explicitly incorporating sex as a core factor in study design and clinical practice, by testing sex‐biassed penetrance of GWAS loci and established T1D genes such as *HLA*, *INS* VNTR, *IL2RA*, *FOXP3*, *CTLA4* and *CLEC16A*, integrating sex‐modified immune signatures, and modelling endocrine and chromosomal effects, will ensure that mechanistic insights are translated into tailored strategies for prediction, monitoring and prevention in both boys and girls at risk.

## FUNDING INFORMATION

Hakon Hakonarson is supported by Institutional Development Funds from The Children's Hospital of Philadelphia to the Center for Applied Genomics, and the Children's Hospital of Philadelphia Endowed Chair in Genomic Research.

## CONFLICT OF INTEREST STATEMENT

The authors declared no potential conflicts of interest with respect to the research, authorship and/or publication of this article.

## PEER REVIEW

The peer review history for this article is available at https://www.webofscience.com/api/gateway/wos/peer‐review/10.1111/dom.70124.

## Supporting information


**Data S1.** Supporting Information.
