# Supplementary material for: Sex as a modifier of genetic risk for type 1 diabetes
Source: Diabetes Obes Metab. 2025 Sep 18;27(12):6857–68. doi: 10.1111/dom.70124 (PMC12587248; doi:10.1111/dom.70124)
Supplement: Supplementary file 1 — Data S1. Supporting Information. [file DOM-27-6857-s001.docx]

**Supplementary Materials**

**Sex as a Modifier of Genetic Risk for Type 1 Diabetes**

Hui-Qi Qu, Hakon Hakonarson

To maintain focus in the main text on sex-specific genetic mechanisms in type 1 diabetes (T1D) and given space limitations in the main article, additional material providing extended immunological and mechanistic context is presented here. Supplementary Text 1 summarizes sex differences in immune cell function in T1D, and Supplementary Text 2 outlines mechanistic models of sex-genotype interaction in immune cells.

**Supplementary Text 1. Sex Differences in Immune Cell Function and T1D**

The autoimmune attack underlying T1D is orchestrated by a defined set of immune cell subsets with proven roles in β-cell destruction^1,2^. CD4⁺ helper T cells, restricted by high-risk *HLA* class II alleles, initiate islet autoimmunity by recognizing β-cell antigens, sustaining insulitis through cytokine production, and providing help to cytotoxic CD8⁺ T cells and B cells^3^. CD8⁺ cytotoxic T cells, particularly those restricted by *HLA-B***39:06* and *HLA-A***24:02*, directly mediate β-cell killing and show sex-biased modulation through androgen receptor signaling^4,5^. Regulatory T cells (Tregs) maintain tolerance, with estrogen- and *FOXP3*-linked pathways influencing their frequency and stability^6,7^. B cells contribute via islet autoantibody production and antigen presentation, with serological profiles showing sex-dependent differences at T1D onset^8,9^. Finally, antigen-presenting cells (dendritic cells and macrophages) regulate initiation and amplification of autoimmunity, with their function shaped by hormone signaling and X-chromosome complement^10,11^. Together, these subsets form the core immune network in which sex exerts modifying effects on T1D risk and progression.

1.1 CD4⁺ T Helper Cells

CD4⁺ T helper cells orchestrate islet autoimmunity by recognizing β-cell peptides presented on *HLA* class II, providing help to cytotoxic T cells and B cells, and secreting cytokines (e.g., IFN-γ, IL-21) that drive insulitis^3^. In T1D-prone NOD mice, female CD4⁺ T cells exhibit enhanced activation and Th1 skewing compared to males, producing higher IFN-γ and lower IL-4 upon T cell receptor (TCR) stimulation. These effects have been linked to estrogen-dependent amplification of IL-12-induced STAT4 phosphorylation and T-bet expression^12^. The resulting Th1/Th17 bias correlates with higher T1D incidence in female NOD mice, while androgen tends to suppress this axis and contribute to protection^12^. The protective effect of androgen administration in female NOD mice contrasts with the male predominance observed in human T1D but is consistent with the broader pattern of male protection seen across many autoimmune diseases^13^. This supports the conclusion that androgen signaling has a genuine immunomodulatory effect that can restrain autoreactive T cell responses^14^.

However, unlike many autoimmune diseases that show a female predominance, T1D exhibits a more complex and variable sex bias across populations and regions. This discrepancy indicates that, in humans, any androgen-mediated restraint of CD4⁺ T cell pathogenicity is overridden by stronger drivers of autoimmunity. T1D is uniquely driven by high-risk *HLA*-class II-restricted CD4⁺ T cell autoimmunity against β-cell antigens established before puberty, prior to the divergence of sex hormone levels. Basal sex differences in human CD4⁺ T cells, characterized by a higher Th1∶Th2 cytokine ratio (e.g., increased IFN-γ:IL-4 production) in men compared with women^15^, can amplify β-cell-specific effector responses. In addition, early-life environmental exposures (e.g., viral infections, microbiome perturbations) can imprint immune trajectories in a way that predisposes to β-cell autoimmunity^16^. Thus, while androgen modulates CD4⁺ T cell polarization in experimental models, their impact on human T1D risk must be considered in the context of dominant genetic and environmental factors.

1.2 CD8⁺ Cytotoxic T Cells
CD8⁺ cytotoxic T lymphocytes recognize β-cell peptides on *HLA* class I and execute killing via perforin, granzyme, and cytokine release^17^. This is particularly relevant given that *HLA*-*B**39:06 and HLA-A**24:02* class I alleles confer increased T1D risk by shaping CD8⁺ T-cell peptide presentation and autoreactivity^4,18^. Intrinsic androgen receptor signaling in CD8⁺ T cells represses effector cytokine IFN-γ via direct AR binding to the *IFNG* locus and promotes an exhaustion-like phenotype with elevated programmed death-1 (PD-1), thereby restraining cytotoxic function in males^5,19^. Extrapolating to T1D, this suggests that AR-mediated restraint could dampen β-cell-specific CTL responses in males, aligning with protective effects of androgen therapy in female NOD mice^12^ and reversal of male protection by castration in NOD mice^20^.

Conversely, Adult males exhibit a lower CD4⁺∶CD8⁺ T-cell ratio, driven by higher CD8⁺ T-cell counts than females^21^, expanding the pool of cytotoxic effectors available for β-cell targeting. The net effect in males therefore reflects two opposing forces: a larger CD8⁺ compartment that increases the likelihood of autoreactive clone expansion, versus androgen-driven functional restraint that limits cytotoxic potency on a per-cell basis. In T1D, the numeric advantage of the male CD8⁺ pool appears to outweigh AR-mediated dampening, contributing to the higher incidence in males despite androgen’s modulatory effects.

Reduced AR signaling in females may allow higher cytotoxic effector gene expression but also faster exhaustion. The PD-1 pathway critically limits CD8⁺ pathogenicity in NOD mice, since PD-1 deficiency accelerates diabetes onset and increases disease penetrance^22^. Estrogen’s impact on autoreactive CD8⁺ responses in human T1D may affect activation, survival, or trafficking during puberty^23^. Moreover, sex chromosome-linked and epigenetic factors shape CD8⁺ T-cell development and responsiveness independently of hormones^24^, though their specific roles in T1D remain to be elucidated.

1.3 Tregs

Tregs (CD4⁺CD25⁺FOXP3⁺) are essential for maintaining self-tolerance by suppressing autoreactive T cells and controlling inflammatory responses, and their impairment contributes to T1D pathogenesis^6^. Estrogens promote Treg differentiation and stability, leading to higher Treg frequency or suppressive capacity in females. For example, physiological estrogen levels upregulate *FOXP3* expression and enhance Treg function in mouse models^24^, and similar effects have been observed *in vitro* with estrogen-driven induction of FOXP3⁺ cells^25^. Because *FOXP3* resides on the X chromosome, X-inactivation patterns or possible escape events could further influence its expression or regulation, potentially contributing to sex-based differences in Treg development or stability^7^. However, context matters: some studies indicate that post-pubertal males have higher circulating Treg frequencies and greater *in vitro* suppressive capacity compared to females, highlighting that hormone-driven effects interact with intrinsic and environmental cues^26,27^.

In T1D, enhanced Treg induction by estrogens might transiently bolster tolerance^28^, yet if autoimmunity is already established early in life, this may be insufficient to prevent progression. Moreover, sex chromosome-linked or epigenetic regulators beyond *FOXP3* likely shape Treg lineage stability or function^29^, but their roles in human T1D remain to be fully elucidated.

1.4 B Cells

B cells contribute to T1D through autoantibody production against islet antigens, antigen presentation to T cells, and cytokine secretion that can amplify pathogenic responses^30^. Females generally mount stronger humoral responses, with higher antibody titers and more robust germinal center reactions^31^. Estrogen signaling drives B cell maturation, class-switch recombination, and somatic hypermutation, which can increase autoantibody diversity and affinity^32^. This enhanced B cell responsiveness in females may underlie sex differences in autoantibody seroconversion rates or kinetics of epitope spreading in preclinical T1D^33^. However, male adolescents show higher prevalence of insulin autoantibodies during puberty than females, indicating a male-skewed B cell autoantibody response that aligns with the male bias in T1D incidence^9^. At the same time, intrinsic differences in B cell subsets or signaling thresholds shaped by sex chromosome complement or epigenetic marks could further modulate antigen presentation efficiency and interaction with T helper cells^24^. Nonetheless, T1D onset patterns reflect additional dominant factors, genetics, early environmental triggers, and hormone dynamics, that modulate B cell involvement in a complex sex-dependent manner.

1.5 Dendritic Cells (DCs)

DCs capture β-cell antigens and present them to T cells, directing the balance between tolerance and activation^34^. Sex hormones influence DC differentiation and function: estrogens enhance DC maturation, upregulate antigen-presentation machinery (e.g., *HLA* class II), and promote proinflammatory cytokine production such as IL-12, which favors Th1 polarization^10^. In contrast, androgens tend to suppress DC maturation and inflammatory cytokine output^35^. These hormone-driven effects can lead to sex-dependent differences in T cell priming: female-biased environments may foster stronger initial activation of autoreactive T cells, whereas male-associated androgen signaling may dampen DC-driven priming. Beyond hormones, sex chromosome-linked genes and epigenetic regulation also shape DC subset distribution or responsiveness^24^, but specific X/Y-linked regulators in DCs relevant to T1D require further study.

Moreover, in human monocyte-derived DC cultures, testosterone enhances IL-12 production and differentiation of inflammatory DCs, suggesting that androgen signaling may paradoxically promote a more proinflammatory DC network in males that amplifies autoreactive CD4⁺ T cell priming and contributes to the male bias in T1D incidence^9^. Fluctuating hormone levels during puberty may transiently alter DC function at critical windows of autoimmunity initiation^36^.

1.6 Monocytes and Macrophages

Monocytes and macrophages detect tissue damage, clear debris, and orchestrate inflammation via cytokine release and phagocytosis^37^. In T1D they contribute to islet inflammation and antigen presentation^38^. Females often exhibit greater responsiveness to Toll-like receptor (TLR) signaling, yielding more robust production of proinflammatory cytokines such as TNF-α and IL-6 upon stimulation, and estrogen can potentiate certain myeloid-cell inflammatory pathways^39,40^. Androgens, conversely, may attenuate TLR-driven cytokine production^41^. AR inhibition alleviated inflammation in experimental autoimmune myocarditis by increasing autophagy in macrophages, suggesting that androgen signaling normally restrains macrophage autophagy and may similarly modulate islet inflammation in T1D^42^. These differences can amplify or restrain the early inflammatory milieu in the pancreas in a sex-specific manner. Furthermore, sex chromosome complement and epigenetic programming can influence monocyte/macrophage differentiation, polarization, and phagocytic or antigen-presenting capacities independently of circulating hormone levels^43,44^. In T1D, heightened innate responsiveness in females could accelerate insulitis once initial β-cell damage occurs, but the ultimate effect depends on interactions with adaptive immunity, genetic risk, and timing relative to hormone fluctuations.

In males, androgen-driven skewing of monocyte/macrophage polarization toward an anti-inflammatory M2-like phenotype^45^ can reduce early proinflammatory responses but may paradoxically impair efficient clearance of apoptotic β-cell debris, leading to sustained antigen presentation and promoting islet autoimmunity. Additionally, Y chromosome-linked genetic factors can alter monocyte recruitment and macrophage turnover in pancreatic tissue, resulting in elevated expression of the anti-apoptotic protein Bcl-2 and a skewed cytokine profile characterized by upregulation of TGF-β1 and downregulation of IL-1β^46^.

In summary, CD4⁺ T helper cells show an estrogen-driven Th1/Th17 bias in NOD mice whereas androgens blunt Th1 skewing, but men’s CD4⁺ pool exhibits a higher basal Th1∶Th2 ratio than women’s; CD8⁺ T cells are more numerous but functionally restrained by androgen signaling in males, whereas female CD8⁺s exhibit higher effector cytokine expression yet reach exhaustion sooner; regulatory T cells benefit from estrogen-driven *FOXP3* upregulation in females and show paradoxically higher frequencies in post-pubertal males; B cells mount more robust germinal center reactions and autoantibody responses in females, with a puberty-linked insulin autoantibody peak in males; DCs generally mature more and secrete more IL-12 under estrogen, with androgens often suppressing this in vivo, yet certain human DC culture studies report testosterone-enhanced IL-12 production, highlighting context-dependent effects; and monocytes and macrophages in females produce greater TNF-α and IL-6 upon TLR stimulation compared with the M2-skewing, Bcl-2- and TGF-β1-driven survival profile seen in males. Altogether, these cell-type-specific sex differences influence when β-cell autoimmunity begins, how aggressively it progresses, and the clinical heterogeneity of T1D.

**Supplementary Text 2. Mechanistic Models of Sex-Genotype Interaction in Immune Cells**

Understanding how sex influences the functional impact of genetic variation in immune cells requires a multilayered exploration of hormone-mediated regulation, sex chromosome complement effects, and epigenetic landscapes. In the context of T1D and other autoimmune conditions, these layers interact with risk variants to produce sex-specific differences in immune responses.

2.1 Hormone-Responsive Regulation of T1D-Associated Genes

Sex steroids engage nuclear receptors, including estrogen receptor (ER) and androgen receptor (AR), which bind specific DNA motifs located in enhancers and promoters. Because these motifs may harbor or flank T1D-associated SNPs, a single nucleotide change can exaggerate or mute the transcriptional response to fluctuating hormone levels^47^. As a result, a given allele may have minimal functional impact under one endocrine condition but exert substantial regulatory effects under another. Estrogen amplifies transcription at clustered estrogen-response elements (EREs) in immune-regulatory loci such as *IL2RA* and *FOXP3*; alleles that weaken ERE affinity reduce inducible transcription and dampen tolerance programs^48,49^. In contrast, AR signaling influences innate immune activity by modulating cytokine production and myeloid cell function. These hormone-dependent effects intersect with innate immune sensing pathways, including AR-mediated modulation of cytokine responses in macrophages and monocytes. Specifically, AR regulates macrophage recruitment and proinflammatory cytokine expression during tissue injury and infection^50^. Direct evidence for AR-mediated modulation of macrophage behavior within pancreatic islets in T1D is currently lacking and requires dedicated investigation. Loss of AR function, whether due to reduced testosterone levels or AR disruption, can amplify innate immune responses, potentially increasing inflammatory signaling in genetically susceptible contexts^50^.

While sex hormones influence many immune pathways, only a subset converge on loci or cell types causally linked to T1D. For example, estrogen-responsive enhancers at *IL2RA* modulate Treg stability^48^, androgen signaling restrains CD8⁺ effector function in *HLA*-restricted contexts^5^, and estrogen-driven *FOXP3* induction enhances tolerance mechanisms^25,49^. These pathways, anchored in well-replicated T1D loci and immune cell functions, provide the clearest mechanistic links between sex and T1D pathogenesis.

2.2 Sex-Chromosome Complement and Gene Dosage

Even in the absence of hormonal cues, the two sexes differ in baseline transcript dosage for dozens of immune genes^51^. Approximately 15 % of X-linked genes escape inactivation in human leucocytes, with an additional ~10-15% showing tissue-variable escape, including *TLR7*, *CD40L*, and *SLC15A4* (*TASL*)^52-54^. Bi-allelic expression in females can magnify either pro-inflammatory or regulatory pathways, amplifying TCR signaling and helper activation in CD4⁺ T cells depending on the locus and cellular context^55^. Conversely, males are hemizygous: a single deleterious allele at an X-linked immune regulator has no buffering partner, heightening functional impact.

Mosaicism further complicates dosage^56^. Skewed X-chromosome inactivation in a female can create T- or B-cell compartments dominated by the higher-expression allele of an escapee, tilting the balance toward either autoimmunity or tolerance without any change in circulating hormones^57^. These dosage effects interact with autosomal risk variants, adding a chromosome-level dimension to gene-gene epistasis.

Additionally, genes on the sex chromosomes influence immune development and function (e.g., X-linked immune genes that may escape inactivation, or Y-linked regulators), creating baseline differences in immune cell subsets or responsiveness that may not align simply with circulating hormone levels. However, the extent and identity of escapees differ markedly between species: in humans, roughly 12-15% of X-linked genes consistently escape X-chromosome inactivation (XCI), with another ~10-15% showing variable escape across tissues, including immune-relevant loci such as Toll-like receptor 7 (*TLR7*), TLR adaptor interacting with endolysosomal *SLC15A4*, and interleukin 1 receptor associated kinase 1 (*IRAK1*)^58^, whereas in mice only ~3-7% of X-linked genes escape XCI and fewer are immune genes^59^. Human and mouse Y chromosomes also differ substantially in gene content and regulatory elements, limiting direct comparison of Y-linked immune modulation. Moreover, although Four Core Genotype (FCG) models have been used to dissect sex chromosome complement effects in several autoimmune contexts (e.g., on C57BL/6 background)^60^, analogous FCG studies in the NOD background are scarce despite the pronounced female bias in NOD diabetes (~60-80% incidence in females vs. ~20-30% in males)^61^. These species- and model-specific limitations underscore the need for more targeted approaches to understand how sex chromosome effects shape autoimmune susceptibility in T1D.

2.3 Sex-Biased Epigenetic Architecture

Genome-wide assays of chromatin accessibility and DNA methylation reveal pervasive sex differences in immune cells (particularly in primary T cells and monocytes) that are independent of circulating hormone at the time of sampling^43,62^. Thousands of enhancers are constitutively more open in one sex^63^, and many coincide with T1D GWAS signals^64^. DNA methylation exhibits widespread autosomal sex dimorphism across human tissues^62^. Wei et al. showed that differential H3K4me3/H3K27me3 patterns at lineage‐defining loci direct Th1 versus Th2/Th17 differentiation, illustrating epigenetic control of helper skewing^65^.

Crucially, hormone receptors preferentially bind pre-opened chromatin^66,67^. However, chromatin accessibility alone does not guarantee receptor occupancy: ER can only engage its response element when sufficient estrogen ligand is present. Therefore, a risk allele located in a “female-open” enhancer resides in an accessible state prior to puberty but will not recruit ER or drive transcription until circulating estrogen levels rise; conversely, in males the same enhancer remains closed and unresponsive unless opened by inflammation or other stimuli^68^. Epigenetic state thus dictates when and in whom hormone-SNP interactions can occur, layering temporal control onto the spatial differences created by sex chromosomes.

Importantly, many sex-biased chromatin states overlap T1D GWAS signals, suggesting that epigenetic architecture does not merely create background differences between the sexes but directly intersects with known susceptibility loci^62,64^. For instance, enhancer accessibility at *IL2RA*, *FOXP3*, and *INS* shows sex-dependent methylation or chromatin openness^65,69^, providing a mechanistic route through which sex modifies established T1D risk genes.

2.4 Convergence of the Three Layers

Hormone sensitivity, X-linked dosage, and epigenetic configuration are not independent; they reinforce or counterbalance one another. The convergence of sex-biased epigenetics, hormone-responsive enhancers, and X-chromosome dosage provides a framework for understanding sex-specific impacts on cytokine signaling and immune checkpoint regulation. For example, *FOXP3* on the X chromosome may be subject to skewed X-chromosome inactivation in female Tregs, resulting in allele-specific dosage variation (dosage layer)^70^. If that enhancer is also hypomethylated (epigenetic layer) and contains a high-affinity ERE (hormone layer), transient estrogen surges can drive super-physiological *FOXP3* expression, strengthening tolerance^71,72^. A SNP that diminishes ER binding at a hypomethylated FOXP3 enhancer in females could blunt estrogen-driven upregulation, whereas in males the closed chromatin state prevents ER access until inflammation or other stimuli open the site. By tracing these multi-layered interactions, we can explain why some genetic effects appear only in one sex, at a particular developmental window, or under a specific inflammatory trigger.

2.5 Timing and Context of Sex Effects in T1D

T1D often manifests in childhood or adolescence, a period when sex hormone concentrations are not yet at stable adult levels and undergo wide fluctuations^73^. Consequently, any immunomodulatory effect of androgens or estrogens is likely transient or too late to prevent established autoreactive processes. Strong genetic predisposition (e.g., high-risk *HLA* alleles) and early-life environmental programming shape immune trajectories well before sex hormones reach adult setpoints^74^. Sex chromosome-linked influences act independently and interact with this immature, variable hormone milieu. Together, early initiation of autoimmunity, unstable sex steroid concentrations around onset, fixed genetic/environmental risk, and inherent sex chromosome effects, underlie the inconsistent sex differences in T1D compared to autoimmune diseases with later onset or different etiologies.

Critically, androgen-mediated restraint in humans is both context- and timing-dependent: prepubertal boys have relatively low testosterone, so early β-cell autoimmunity can initiate unopposed, and the subsequent peripubertal androgen surge may speculatively arrive too late to prevent clinical onset^75^. Moreover, inter-individual differences in AR signaling capacity and Y-linked immunoregulatory factors can blunt the magnitude of androgen’s brake, such that in many males even a competent androgen response is insufficient to fully counteract the strong *HLA*-driven autoimmune drive^76^.

**Supplementary References:**

1. Lehuen A, Diana J, Zaccone P, Cooke A. Immune cell crosstalk in type 1 diabetes. *Nature Reviews Immunology.* 2010;10(7):501-513.

2. Navegantes KC, de Souza Gomes R, Pereira PAT, Czaikoski PG, Azevedo CHM, Monteiro MC. Immune modulation of some autoimmune diseases: the critical role of macrophages and neutrophils in the innate and adaptive immunity. *Journal of translational medicine.* 2017;15:1-21.

3. Espinosa-Carrasco G, Le Saout C, Fontanaud P, et al. CD4+ T helper cells play a key role in maintaining diabetogenic CD8+ T cell function in the pancreas. *Frontiers in immunology.* 2018;8:2001.

4. Yeo L, Pujol-Autonell I, Baptista R, et al. Circulating β cell-specific CD8(+) T cells restricted by high-risk HLA class I molecules show antigen experience in children with and at risk of type 1 diabetes. *Clinical and experimental immunology.* 2020;199(3):263-277.

5. Kwon H, Schafer JM, Song NJ, et al. Androgen conspires with the CD8(+) T cell exhaustion program and contributes to sex bias in cancer. *Sci Immunol.* 2022;7(73):eabq2630.

6. Visperas A, Vignali DA. Are regulatory T cells defective in type 1 diabetes and can we fix them? *The Journal of Immunology.* 2016;197(10):3762-3770.

7. Fairweather D, Beetler DJ, McCabe EJ, Lieberman SM. Mechanisms underlying sex differences in autoimmunity. *The Journal of clinical investigation.* 2024;134(18).

8. Pihoker C, Gilliam LK, Hampe CS, Lernmark Ak. Autoantibodies in Diabetes. *Diabetes.* 2005;54(suppl_2):S52-S61.

9. Williams AJK, Norcross AJ, Dix RJ, Gillespie KM, Gale EAM, Bingley PJ. The prevalence of insulin autoantibodies at the onset of Type 1 diabetes is higher in males than females during adolescence. *Diabetologia.* 2003;46(10):1354-1356.

10. Mackern-Oberti JP, Jara EL, Riedel CA, Kalergis AM. Hormonal modulation of dendritic cells differentiation, maturation and function: implications for the initiation and progress of systemic autoimmunity. *Archivum immunologiae et therapiae experimentalis.* 2017;65(2):123-136.

11. Price JD, Tarbell KV. The role of dendritic cell subsets and innate immunity in the pathogenesis of type 1 diabetes and other autoimmune diseases. *Frontiers in immunology.* 2015;6:288.

12. Bao M, Yang Y, Jun HS, Yoon JW. Molecular mechanisms for gender differences in susceptibility to T cell-mediated autoimmune diabetes in nonobese diabetic mice. *J Immunol.* 2002;168(10):5369-5375.

13. Voskuhl R. Sex differences in autoimmune diseases. *Biology of sex differences.* 2011;2:1-21.

14. Gubbels Bupp MR, Jorgensen TN. Androgen-induced immunosuppression. *Frontiers in immunology.* 2018;9:370132.

15. Giron-Gonzalez J, Moral FJ, Elvira J, et al. Consistent production of a higher TH1: TH2 cytokine ratio by stimulated T cells in men compared with women. *European journal of endocrinology.* 2000;143(1):31-36.

16. Craig ME, Kim KW, Isaacs SR, et al. Early-life factors contributing to type 1 diabetes. *Diabetologia.* 2019;62:1823-1834.

17. Mallone R, Martinuzzi E, Blancou P, et al. CD8+ T-cell responses identify β-cell autoimmunity in human type 1 diabetes. *Diabetes.* 2007;56(3):613-621.

18. Schloss J, Ali R, Racine JJ, Chapman HD, Serreze DV, DiLorenzo TP. HLA-B*39:06 Efficiently Mediates Type 1 Diabetes in a Mouse Model Incorporating Reduced Thymic Insulin Expression. *J Immunol.* 2018;200(10):3353-3363.

19. Hussain T, Kallies A, Vasanthakumar A. Sex-bias in CD8(+) T-cell stemness and exhaustion in cancer. *Clinical & translational immunology.* 2022;11(8):e1414.

20. Lee J, Yurkovetskiy LA, Reiman D, et al. Androgens contribute to sex bias of autoimmunity in mice by T cell-intrinsic regulation of Ptpn22 phosphatase expression. *Nature Communications.* 2024;15(1):7688.

21. Uppal SS, Verma S, Dhot PS. Normal values of CD4 and CD8 lymphocyte subsets in healthy indian adults and the effects of sex, age, ethnicity, and smoking. *Cytometry Part B, Clinical cytometry.* 2003;52(1):32-36.

22. Fife BT, Guleria I, Gubbels Bupp M, et al. Insulin-induced remission in new-onset NOD mice is maintained by the PD-1-PD-L1 pathway. *J Exp Med.* 2006;203(12):2737-2747.

23. Lang TJ. Estrogen as an immunomodulator. *Clinical immunology.* 2004;113(3):224-230.

24. Bhattacharya S, Sadhukhan D, Saraswathy R. Role of sex in immune response and epigenetic mechanisms. *Epigenetics & Chromatin.* 2024;17(1):1.

25. Gourdy P, Bourgeois EA, Levescot A, et al. Estrogen Therapy Delays Autoimmune Diabetes and Promotes the Protective Efficiency of Natural Killer T-Cell Activation in Female Nonobese Diabetic Mice. *Endocrinology.* 2016;157(1):258-267.

26. Robinson GA, Peng J, Peckham H, et al. Investigating sex differences in T regulatory cells from cisgender and transgender healthy individuals and patients with autoimmune inflammatory disease: a cross-sectional study. *The Lancet Rheumatology.* 2022;4(10):e710-e724.

27. Afshan G, Afzal N, Qureshi S. CD4+CD25(hi) regulatory T cells in healthy males and females mediate gender difference in the prevalence of autoimmune diseases. *Clinical laboratory.* 2012;58(5-6):567-571.

28. Polanczyk MJ, Hopke C, Vandenbark AA, Offner H. Estrogen‐mediated immunomodulation involves reduced activation of effector T cells, potentiation of Treg cells, and enhanced expression of the PD‐1 costimulatory pathway. *Journal of neuroscience research.* 2006;84(2):370-378.

29. Shepherd R, Cheung AS, Pang K, Saffery R, Novakovic B. Sexual Dimorphism in Innate Immunity: The Role of Sex Hormones and Epigenetics. *Front Immunol.* 2020;11:604000.

30. Smith MJ, Simmons KM, Cambier JC. B cells in type 1 diabetes mellitus and diabetic kidney disease. *Nature Reviews Nephrology.* 2017;13(11):712-720.

31. Fink AL, Klein SL. The evolution of greater humoral immunity in females than males: implications for vaccine efficacy. *Current opinion in physiology.* 2018;6:16-20.

32. Asaba J, Bandyopadhyay M, Kindy M, Dasgupta S. Estrogen receptor signal in regulation of B cell activation during diverse immune responses. *The international journal of biochemistry & cell biology.* 2015;68:42-47.

33. Wang Y-n, Li R, Huang Y, et al. The role of B cells in the pathogenesis of type 1 diabetes. *Frontiers in Immunology.* 2024;15:1450366.

34. Turley S, Poirot L, Hattori M, Benoist C, Mathis D. Physiological β cell death triggers priming of self-reactive T cells by dendritic cells in a type-1 diabetes model. *The Journal of experimental medicine.* 2003;198(10):1527-1537.

35. Koh YT, Gray A, Higgins SA, Hubby B, Kast WM. Androgen ablation augments prostate cancer vaccine immunogenicity only when applied after immunization. *The Prostate.* 2009;69(6):571-584.

36. Ucciferri CC, Dunn SE. Effect of puberty on the immune system: Relevance to multiple sclerosis. *Frontiers in pediatrics.* 2022;10:1059083.

37. Ginhoux F, Jung S. Monocytes and macrophages: developmental pathways and tissue homeostasis. *Nature Reviews Immunology.* 2014;14(6):392-404.

38. Willcox A, Richardson S, Bone A, Foulis A, Morgan N. Analysis of islet inflammation in human type 1 diabetes. *Clinical & Experimental Immunology.* 2009;155(2):173-181.

39. Kovats S. Estrogen receptors regulate innate immune cells and signaling pathways. *Cell Immunol.* 2015;294(2):63-69.

40. Cunningham MA, Naga OS, Eudaly JG, Scott JL, Gilkeson GS. Estrogen receptor alpha modulates toll-like receptor signaling in murine lupus. *Clinical Immunology.* 2012;144(1):1-12.

41. Traish A, Bolanos J, Nair S, Saad F, Morgentaler A. Do Androgens Modulate the Pathophysiological Pathways of Inflammation? Appraising the Contemporary Evidence. *J Clin Med.* 2018;7(12).

42. Ma WH, Zhang XG, Guo LL, et al. Androgen receptor inhibition alleviated inflammation in experimental autoimmune myocarditis by increasing autophagy in macrophages. *European review for medical and pharmacological sciences.* 2021;25(10):3762-3771.

43. Chlamydas S, Markouli M, Strepkos D, Piperi C. Epigenetic mechanisms regulate sex-specific bias in disease manifestations. *Journal of Molecular Medicine.* 2022;100(8):1111-1123.

44. Laffont S, Rouquié N, Azar P, et al. X-Chromosome complement and estrogen receptor signaling independently contribute to the enhanced TLR7-mediated IFN-α production of plasmacytoid dendritic cells from women. *The Journal of Immunology.* 2014;193(11):5444-5452.

45. Becerra-Díaz M, Strickland AB, Keselman A, Heller NM. Androgen and Androgen Receptor as Enhancers of M2 Macrophage Polarization in Allergic Lung Inflammation. *J Immunol.* 2018;201(10):2923-2933.

46. Charchar FJ, Bloomer LD, Barnes TA, et al. Inheritance of coronary artery disease in men: an analysis of the role of the Y chromosome. *Lancet (London, England).* 2012;379(9819):915-922.

47. Carroll JS, Meyer CA, Song J, et al. Genome-wide analysis of estrogen receptor binding sites. *Nat Genet.* 2006;38(11):1289-1297.

48. Afanasyeva MA, Putlyaeva LV, Demin DE, et al. The single nucleotide variant rs12722489 determines differential estrogen receptor binding and enhancer properties of an IL2RA intronic region. *PLoS One.* 2017;12(2):e0172681.

49. Polanczyk MJ, Carson BD, Subramanian S, et al. Cutting edge: estrogen drives expansion of the CD4+CD25+ regulatory T cell compartment. *J Immunol.* 2004;173(4):2227-2230.

50. Lai JJ, Lai KP, Zeng W, Chuang KH, Altuwaijri S, Chang C. Androgen receptor influences on body defense system via modulation of innate and adaptive immune systems: lessons from conditional AR knockout mice. *Am J Pathol.* 2012;181(5):1504-1512.

51. Klein SL, Flanagan KL. Sex differences in immune responses. *Nature Reviews Immunology.* 2016;16(10):626-638.

52. Tukiainen T, Villani A-C, Yen A, et al. Landscape of X chromosome inactivation across human tissues. *Nature.* 2017;550(7675):244-248.

53. Mousavi MJ, Mahmoudi M, Ghotloo S. Escape from X chromosome inactivation and female bias of autoimmune diseases. *Molecular Medicine.* 2020;26:1-20.

54. Carrel L, Willard HF. X-inactivation profile reveals extensive variability in X-linked gene expression in females. *Nature.* 2005;434(7031):400-404.

55. Youness A, Cenac C, Faz-López B, et al. TLR8 escapes X chromosome inactivation in human monocytes and CD4(+) T cells. *Biol Sex Differ.* 2023;14(1):60.

56. Spolarics Z, Peña G, Qin Y, Donnelly RJ, Livingston DH. Inherent X-linked genetic variability and cellular mosaicism unique to females contribute to sex-related differences in the innate immune response. *Frontiers in Immunology.* 2017;8:1455.

57. Bianchi I, Lleo A, Gershwin ME, Invernizzi P. The X chromosome and immune associated genes. *Journal of autoimmunity.* 2012;38(2-3):J187-J192.

58. Huret C, Ferrayé L, David A, et al. Altered X-chromosome inactivation predisposes to autoimmunity. *Science advances.* 2024;10(18):eadn6537.

59. Balaton BP, Fornes O, Wasserman WW, Brown CJ. Cross-species examination of X-chromosome inactivation highlights domains of escape from silencing. *Epigenetics & Chromatin.* 2021;14(1):12.

60. Arnold AP. Four Core Genotypes and XY* mouse models: Update on impact on SABV research. *Neuroscience & Biobehavioral Reviews.* 2020;119:1-8.

61. Aldrich VR, Hernandez-Rovira BB, Chandwani A, Abdulreda MH. NOD Mice-Good Model for T1D but Not Without Limitations. *Cell transplantation.* 2020;29:963689720939127.

62. Grant OA, Wang Y, Kumari M, Zabet NR, Schalkwyk L. Characterising sex differences of autosomal DNA methylation in whole blood using the Illumina EPIC array. *Clinical Epigenetics.* 2022;14(1):62.

63. Sugathan A, Waxman DJ. Genome-wide analysis of chromatin states reveals distinct mechanisms of sex-dependent gene regulation in male and female mouse liver. *Molecular and cellular biology.* 2013;33(18):3594-3610.

64. Jerram ST, Dang MN, Leslie RD. The role of epigenetics in type 1 diabetes. *Current diabetes reports.* 2017;17:1-11.

65. Wei G, Wei L, Zhu J, et al. Global mapping of H3K4me3 and H3K27me3 reveals specificity and plasticity in lineage fate determination of differentiating CD4+ T cells. *Immunity.* 2009;30(1):155-167.

66. Rawłuszko-Wieczorek AA, Romanowska K, Nowicki M. Chromatin modifiers-Coordinators of estrogen action. *Biomedicine & Pharmacotherapy.* 2022;153:113548.

67. Hewitt SC, Korach KS. Estrogen receptors: new directions in the new millennium. *Endocrine reviews.* 2018;39(5):664-675.

68. Bayarsaihan D. Epigenetic mechanisms in inflammation. *Journal of dental research.* 2011;90(1):9-17.

69. Pahkuri S, Ekman I, Vandamme C, et al. DNA methylation differences within INS, PTPN22 and IL2RA promoters in lymphocyte subsets in children with type 1 diabetes and controls. *Autoimmunity.* 2023;56(1):2259118.

70. Tommasini A, Ferrari S, Moratto D, et al. X-chromosome inactivation analysis in a female carrier of FOXP3 mutation. *Clinical and experimental immunology.* 2002;130(1):127-130.

71. Lal G, Bromberg JS. Epigenetic mechanisms of regulation of Foxp3 expression. *Blood.* 2009;114(18):3727-3735.

72. Adurthi S, Kumar MM, Vinodkumar HS, et al. Oestrogen Receptor-α binds the FOXP3 promoter and modulates regulatory T-cell function in human cervical cancer. *Sci Rep.* 2017;7(1):17289.

73. Kordonouri O, Hartmann R, Deiss D, Wilms M, Grüters-Kieslich A. Natural course of autoimmune thyroiditis in type 1 diabetes: association with gender, age, diabetes duration, and puberty. *Archives of disease in childhood.* 2005;90(4):411-414.

74. Dahlquist G. Can we slow the rising incidence of childhood-onset autoimmune diabetes? The overload hypothesis. *Diabetologia.* 2006;49(1):20-24.

75. Peltonen EJ, Veijola R, Ilonen J, et al. What is the role of puberty in the development of islet autoimmunity and progression to type 1 diabetes? *European Journal of Epidemiology.* 2023;38(6):689-697.

76. Case LK, Wall EH, Dragon JA, et al. The Y chromosome as a regulatory element shaping immune cell transcriptomes and susceptibility to autoimmune disease. *Genome research.* 2013;23(9):1474-1485.
